# Vibration Analysis of Porous Cu-Si Microcantilever Beams in Fluids Based on Modified Couple Stress Theory

**DOI:** 10.3390/nano14131144

**Published:** 2024-07-03

**Authors:** Jize Jiang, Feixiang Tang, Siyu He, Fang Dong, Sheng Liu

**Affiliations:** 1 Key Laboratory of Transients in Hydraulic Machinery, Ministry of Education, School of Power and Mechanical Engineering, Wuhan University, Wuhan 430072, China; 2021302191690@whu.edu.cn; 2 China-EU Insititute for Clean and Renewable Energy, Huazhong University of Science & Technology, Wuhan 430074, China; hsy_hust@hust.edu.cn; 3 The Institute of Technological Sciences, Wuhan University, Wuhan 430072, China; dongfang@whu.edu.cn

**Keywords:** vibration, porosity, scale effects, microcantilever beams, quality factor

## Abstract

The vibrations in functionally graded porous Cu-Si microcantilever beams are investigated based on physical neutral plane theory, modified coupled stress theory, and scale distribution theory (MCST&SDT). Porous microcantilever beams define four pore distributions. Considering the physical neutral plane theory, the material properties of the beams are computed through four different power-law distributions. The material properties of microcantilever beams are corrected by scale effects based on modified coupled stress theory. Considering the fluid driving force, the amplitude-frequency response spectra and resonant frequencies of the porous microcantilever beam in three different fluids are obtained based on the Euler–Bernoulli beam theory. The quality factors of porous microcantilever beams in three different fluids are derived by estimating the equation. The computational analysis shows that the presence of pores in microcantilever beams leads to a decrease in Young’s modulus. Different pore distributions affect the material properties to different degrees. The gain effect of the scale effect is weakened, but the one-dimensional temperature field and amplitude-frequency response spectra show an increasing trend. The quality factor is decreased by porosity, and the degree of influence of porosity increases as the beam thickness increases. The gradient factor n has a greater effect on the resonant frequency. The effect of porosity on the resonant frequency is negatively correlated when the gradient factor is small (n<1) but positively correlated when the gradient factor is large (n>1).

## 1. Introduction

Micro-electro-mechanical systems (MEMS) are widely used in aerospace, biochemical testing, and environmental monitoring [1,2,3,4,5]. The functionally graded microbeam (FGM), as a typical structure in MEMS, plays an irreplaceable role in many high-precision fields, including electronics, mechanics, materials, automation, physics, chemistry, biology, and other disciplines. It has shown a wide range of applications in consumer electronics, industrial control, medical science and technology, communications, and national defense [6]. Functionally graded materials are uniquely suited for tuning material properties. Superior material properties can lead to great performance gains in MEMS devices. Compared with traditional materials, functionally graded materials have many excellent properties [7,8,9], such as higher strength, more sensitive resonance properties, higher temperature resistance, better corrosion resistance, etc. In a study of microcantilever beams, the pore structure can effectively reduce the material density and Young’s modulus [10]. This improves the vibration characteristics, durability, and mechanical properties of the beam. Directional control of the microcantilever beam’s stiffness and deflection can be achieved by rationally designing the pore structure to meet specific engineering requirements.

In recent years, many scholars at home and abroad have devoted themselves to researching vibrations in functional gradient cantilever beams. Hichen Bellifa et al. [11] proposed a new first-order shear deformation theory and the concept of a physical neutral surface to study the dynamic behavior of the FGM plate. Liang et al. [12] established a vibration model of the FGM cantilever beam based on the modified dipole stress theory and found that the intrinsic frequency of the microcantilever beam gradually drifted toward a high frequency with an increase in the Si component and gradually drifted to higher frequencies. Kiracofe et al. [13] investigated the photothermal excitation of microcantilever beams in fluids and illustrated the effect of the geometry of microcantilever beams on the photothermal response. Galerkin’s method is a numerical computational method for solving mechanical differential equations [14]. The solution to the Euler–Bernoulli beam differential equation can be obtained by weighting the solutions of the trial function series. Bao et al. [15] have investigated the vibration and stability of a rotating viscoelastic conical shaft using the Laplace transform and Galerkin’s methods. The intrinsic frequency and modal damping were calculated. Soltani et al. [16] proposed an improved approach based on the power series expansions to exactly evaluate the static and buckling stiffness matrices for the linear stability analysis of axially functionally graded (AFG) Timoshenko beams with variable cross-sections and fixed–free boundary conditions. For microcantilever beams in fluids, considering photothermal excitation, D. Ramos et al. [17] established the one-dimensional heat transfer equations for gold-silicon cantilever beams and their vibrational response in water. Gu et al. [18] investigated the one-dimensional temperature field distributions and frequency responses of three FGM microbeam structures in different fluids based on the Euler–Bernoulli beam theory. When considering porosity, the vibration characteristics of microcantilever beams change significantly. Gao et al. [19] considered the static and dynamic response of functionally graded micro/nanoplates as a basis for developing functionally graded MEMS devices. They concluded that the increase in porosity decreases the critical buckling load and reduces the beam stiffness, and they designed the minimum critical buckling load in the porosity distribution. Zenkour et al. [20] investigated the bending response of porous functionally graded monolayer and sandwich thick rectangular plates using quasi-3D shear deformation. The effects of power-law exponents and porosity coefficients are emphasized.

When the size of the material structure is reduced to the micrometer level, many experimental phenomena [21,22,23] show that its mechanical behavior and material properties have noticeable differences from those at the macroscopic scale [24,25]. For micrometer scale systems, the modified couple stress theory is the most commonly studied theory [26]. The scale effect is the method used in this theory. At this time, it is of great significance to study the scale effect correction of the theoretical model of the functionally graded microcantilever beam. Lam et al. [27] found a sudden increase in the equivalent stiffness of 20-μm thick cantilever beams when they carried out bending experiments on epoxy resin cantilever beams with thicknesses ranging from 20 to 115 μm using the nanoindentation technique. Later, Lei et al. [28] found the scale effect on the natural frequency of metal microbeam vibration when studying the vibration response in 2~15 μm nickel-silicon cantilever beams, proving that the scale effect has a significant influence on the mechanical properties of the microstructures. Tang et al. [29], in using Kirchhoff’s thin-plate theory to study the bending, buckling, and vibration in FGMs by considering the microscopic scale effect, they proposed to use the concept of the equivalent bending stiffness of the microbeams to describe the scale effect. They obtained the expressions for the modification of Young’s modulus in the theoretical model under the microscopic scale effects. Shi et al. [30] conducted a vibration analysis of Kirchhoff thin plates with four pore distributions based on the modified couple stress theory.

In this paper, we innovatively introduce four pore distributions into cantilever beams based on the study of the forced vibrations of microcantilever beams in different fluids under scale effect. Based on the physical neutral plane theory, we have investigated the properties of Cu-Si microcantilever beam materials using the power-law distribution method. The one-dimensional temperature field of a microcantilever beam subjected to laser excitation is obtained. Based on the Euler–Bernoulli theory, the vibration response solutions of microcantilever beams subjected to thermally driven and hydrodynamic conditions are given computationally using Galerkin’s method. The effects of porosity, scale effect, gradient factor, fluid properties, and geometry on Young’s modulus, resonant frequency, and quality factor of the beam are also discussed by numerical analysis. The above studies are relevant for mass sensing and fluid characterization related to cantilever beam structures [31].

## 2. Theoretical Analysis

### 2.1. Power-Law Model of Porous FGM Building

Functionally graded microcantilever beams are made of a composite of two materials. The upper surface material is Cu, and the lower surface material is Si. In the *z*-axis direction, the material properties vary uniformly from bottom to top. There are no visible interfaces inside the cantilever beam. The materials are tightly bonded and have almost no residual internal stresses.

**Figure 1:**
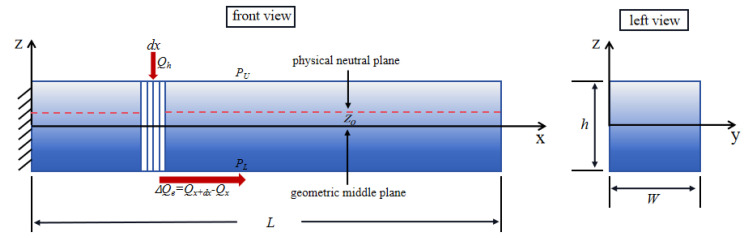
Power-law distribution and one-dimensional heat transfer model of FGM microcantilever beams.

Based on the physical neutral plane theory, a power law theory model for microcantilever beams can be developed, as shown in Figure 1. Due to inhomogeneity in the functionally graded material, there is some deviation of the physical neutral plane from the geometric midplane. In order to avoid the material tension–bending coupling, we use the parameter z0 to correct the physical neutral plane.

**Figure 2:**
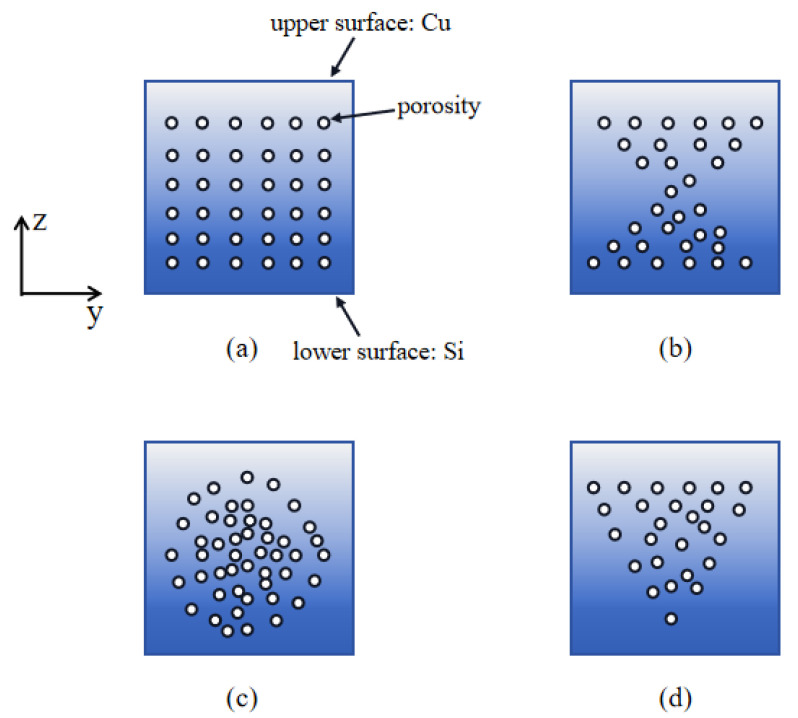
Four pore distributions defined at the microcantilever beam cross-section. (**a**) Even distribution; (**b**) X-type distribution; (**c**) O-type distribution; (**d**) V-type distribution.

Based on the physical neutral plane theory, the power-law distribution and the one-dimensional heat transfer theory model of the FGM microcantilever beam are given in Figure 1. In Figure 2, we define four pore distributions in the *y*-*z* axis cross-section direction of the microcantilever beam. These four distributions are (a) even distribution, (b) X-type distribution, (c) O-type distribution, and (d) V-type distribution.

Considering the four pore distributions, the power-law distribution equation for the material properties of the FGM microcantilever beam 30 can be given as

Even distribution:

(1) $$P\left(z\right)=P_{2}+\left(P_{1}-P_{2}\right){\left(\frac{1}{2}+\frac{z}{h}\right)}^{n}-\frac{\alpha }{2}\left(P_{1}+P_{2}\right)$$

X-type distribution:

(2) $$P\left(z\right)=P_{2}+\left(P_{1}-P_{2}\right){\left(\frac{1}{2}+\frac{z}{h}\right)}^{n}-\frac{\alpha }{2}\left(P_{1}+P_{2}\right)\frac{2\left|z\right|}{h}$$

O-type distribution:

(3) $$P\left(z\right)=P_{2}+\left(P_{1}-P_{2}\right){\left(\frac{1}{2}+\frac{z}{h}\right)}^{n}-\frac{\alpha }{2}\left(P_{1}+P_{2}\right)\left(1-\frac{2\left|z\right|}{h}\right)$$

V-type distribution:

(4) $$P\left(z\right)=P_{2}+\left(P_{1}-P_{2}\right){\left(\frac{1}{2}+\frac{z}{h}\right)}^{n}-\frac{\alpha }{2}\left(P_{1}+P_{2}\right)\left(\frac{1}{2}+\frac{z}{h}\right)$$

where P(z) represents the material properties at a certain z value along the thickness direction. $P_{1}$ represents the material properties of Cu and $P_{2}$ represents the material properties of Si, such as Young’s modulus E, density ρ, scale constant l, Poisson’s ratio v, thermal conductivity κ, specific heat capacity C, and coefficient of thermal expansion β. n  is the gradient factor. This value represents the material percentage of Cu and Si in the FGM microcantilever beam. When n=0, the microcantilever beam consists of pure Cu; when n=∞, the microcantilever beam consists of pure Si. α is the porosity inside the microcantilever beam. h is the thickness of the microcantilever beam.

The expressions for the distribution of material properties of four porous microcantilever beams are known. We can express the equivalent scale constant $l_{eff}$ and the equivalent Poisson’s ratio $v_{eff}$ as follows:

(5) $$l_{eff}=\int_{-\frac{h}{2}}^{\frac{h}{2}}\frac{l(z)}{h}dz$$

(6) $$v_{eff}=\int_{-\frac{h}{2}}^{\frac{h}{2}}\frac{v(z)}{h}dz$$

Based on the modified couple stress theory, we use the scale effect to modify Young’s modulus of the micrometer cantilever beam. From the literature [29], the equivalent bending stiffness is used to describe the scale effect correction to Young’s modulus of Kirchhoff thin plates. Similarly, the modified expression for the scale effect of Young’s modulus of the microcantilever beam is as follows:

(7) $$E^{\prime }\left(z\right)=E\left(z\right)+\frac{6E\left(z\right)l_{eff}^{2}\left(1-v_{eff}\right)}{h^{2}}$$

(8) $$E_{eff}=\int_{-\frac{h}{2}}^{\frac{h}{2}}\frac{E^{\prime }\left(z\right)}{h}dz$$

Based on the physical neutral plane theory, the physical neutral plane is the plane where the stress and strain are zero in the pure bending of the material. For isotropic materials, the physical neutral plane coincides with the geometric midplane. In FGM microcantilever beams, the deviation z_0_ of the physical neutral plane from the geometric midplane can be expressed as:

(9) $$z_{0}=\frac{\int_{-\frac{h}{2}}^{\frac{h}{2}}zE^{\prime }\left(z\right)dz}{E_{eff}}$$

### 2.2. One-Dimensional Temperature Field

In a fluid environment, the FGM microcantilever beam is excited by the laser to produce an internal temperature distribution field. As in the theoretical model of Figure 1, the microcantilever beam is of length L, width W, and height h. Since the thermal diffusion length is much larger than the beam thickness and the laser spot diameter is about the same as the width of the beam, the temperature field is assumed to be constant along the *y*- and *z*-axis directions. Thus, the one-dimensional heat transfer equation for a functional gradient beam can be established.

Following Equations (1)–(6), we can express the equivalent material parameters required for a one-dimensional temperature field as follows:

(10) $$\left[\begin{array}{c} \kappa_{eff}\\ \rho_{eff}\\ C_{eff} \end{array}\right]=\left[\begin{array}{c} \int_{-\frac{h}{2}}^{\frac{h}{2}}\frac{\kappa (z)}{h}dz\\ \int_{-\frac{h}{2}}^{\frac{h}{2}}\frac{\rho (z)}{h}dz\\ \int_{-\frac{h}{2}}^{\frac{h}{2}}\frac{C(z)}{h}dz \end{array}\right]$$

In the model in Figure 1, a thermally conductive microelement dx is taken. Under the laser excitation, its heat increment is $\Delta Q_{e}$. The temperature of the microelement dx is T. The temperature of the fluid environment is $T_{hydro}$. The heat transfer coefficient between the microcantilever beam and the fluid environment is γ, and the heat increment of the heat transfer is $Q_{h}$. After laser heating and fluid heat exchange, the energy storage term of the microelement is $Q_{st}$. From the theory of heat conduction and conservation of energy, the one-dimensional temperature field equation for a microcantilever beam 18 is derived as follows:

(11) $$\Delta Q_{e}=W\cdot \kappa_{eff}\cdot \frac{\partial^{2}T}{\partial x^{2}}dx$$

(12) $$Q_{h}=2\gamma \cdot \left(W+h\right)\left(T-T_{hydro}\right)dx$$

(13) $$Q_{st}=\rho_{eff}C_{eff}Wh\cdot \frac{\partial T}{\partial t}dx$$

(14) $$Q_{st}=\Delta Q_{e}-Q_{h}$$

By substituting Equations (11)–(13) into Equation (14), we can obtain the one-dimensional temperature field control equation for the FGM microcantilever beam as follows:

(15) $$\frac{\partial \left(T-T_{hydro}\right)}{\partial t}=K\frac{\partial^{2}\left(T-T_{hydro}\right)}{\partial x^{2}}-R\left(T-T_{hydro}\right)$$

where K and R are simplifying parameters.

(16) $$K=\frac{\kappa_{eff}}{\rho_{eff}C_{eff}}$$

(17) $$R=\frac{2\gamma \cdot \left(W+h\right)}{W\rho_{eff}C_{eff}}$$

Equation (15) is Fourier transformed and $∆\hat{T}$ is defined as the temperature increment in the frequency domain. The thermal boundary conditions are first determined as

(18) $$∆\hat{T}\left(x_{0}^{-}\right)=∆\hat{T}\left(x_{0}^{+}\right)$$

(19) $${\left.\frac{\partial ∆\hat{T}}{\partial x}\right|}_{x=x_{0}^{+}}-{\left.\frac{\partial ∆\hat{T}}{\partial x}\right|}_{x=x_{0}^{-}}=-\frac{\lambda P_{0}}{W\kappa_{eff}}$$

(20) $${\left.∆\hat{T}\right|}_{x=0}=0,\;{\left.\frac{\partial ∆\hat{T}}{\partial x}\right|}_{x=L}=-\frac{\gamma h}{\kappa_{eff}}∆\hat{T}$$

where Equation (18) indicates that the temperature on the beam satisfies the continuity condition at $x=x_{0}$ for laser loading. Equation (19) indicates that the thermos-fluid temperature satisfies the jump condition at $x=x_{0}$ for laser loading. λ represents the absorption coefficient of laser energy by the microcantilever beam. $P_{0}$ is the laser power. Equation (20) represents the heat transfer boundary conditions for a cantilever beam. In Equation (20), we assume that there is no heat flow loss at the fixed end, and the free end satisfies the heat convection condition.

The frequency domain generalized solution of Equation (15) after Fourier transform can be expressed as:

(21) $$\left\{\begin{array}{c} ∆\hat{T}\left(x,\omega \right)=H_{1}e^{rx}+H_{2}e^{-rx},\left(x<x_{0}\right)\\ ∆\hat{T}\left(x,\omega \right)=H_{3}e^{rx}+H_{4}e^{-rx},\left(x\geq x_{0}\right) \end{array}\right.$$

where ω is the circular frequency. r is the simplifying factor in the complex plane. Their expressions are as follows:

(22) ω=2πf

(23) $$r=\sqrt{\frac{R+\sqrt{R^{2}+\omega^{2}}}{2K}}+i\sqrt{\frac{-R+\sqrt{R^{2}+\omega^{2}}}{2K}}$$

The generalization coefficients $H_{1}\sim H_{4}$ in Equation (21) are determined by the boundary conditions of the Fourier transform. From Equations (18)–(20) the derivation can be made as follows:

(24) $$\left\{\begin{array}{c} \begin{array}{c} \begin{array}{c} H_{1}=\frac{\lambda P_{0}}{W\kappa_{eff}}\times \frac{e^{-rx_{0}}\cdot \left[e^{2rL}\left(r+\frac{\gamma h}{\kappa_{eff}}\right)+e^{2rx_{0}}\left(r-\frac{\gamma h}{\kappa_{eff}}\right)\right]}{2r\left[e^{2rL}\left(r+\frac{\gamma h}{\kappa_{eff}}\right)+\left(r-\frac{\gamma h}{\kappa_{eff}}\right)\right]}\\ H_{2}=-H_{1}\\ H_{3}=\frac{\lambda P_{0}}{W\kappa_{eff}}\times \frac{e^{-rx_{0}}\cdot \left(e^{2rx_{0}}-1\right)\left(r-\frac{\gamma h}{\kappa_{eff}}\right)}{2r\left[e^{2rL}\left(r+\frac{\gamma h}{\kappa_{eff}}\right)+\left(r-\frac{\gamma h}{\kappa_{eff}}\right)\right]}\\ H_{4}=\frac{H_{3}\cdot e^{2rL}\left(r+\frac{\gamma h}{\kappa_{eff}}\right)}{\left(r-\frac{\gamma h}{\kappa_{eff}}\right)} \end{array} \end{array} \end{array}\right.$$

### 2.3. Driving Forces and Dynamic Response

The FGM microcantilever beam is subjected to the combined action of the photothermal driving force $F_{drive}$ and the hydrodynamic force $F_{hydro}$ when it is vibrated by laser excitation in a fluid.

(25) $$F_{eff}\left(x,\omega \right)=F_{drive}\left(x,\omega \right)+F_{hydro}\left(x,\omega \right)$$

Due to the different material properties of the functionally graded beams along the *z*-axis, the beams develop asymptotic axial stresses in the thickness direction. From the thermoelasticity theory, the thermal stress distribution along the z direction and the bending moment distribution along the x direction of the FGM microcantilever beam can be obtained as follows:

(26) $$\sigma \left(z\right)=E\left(z\right)\beta \left(z\right)\Delta \hat{T}\left(x,\omega \right)$$

(27) $$M\left(x,\omega \right)=W\int_{-\frac{h}{2}}^{\frac{h}{2}}\sigma \left(z\right)\left(z-z_{0}\right)dz$$

Based on the classical beam theory, there is a double differential relationship between the axial bending moment and the shear driving force. Then, $F_{drive}$ can be derived as follows:

(28) $$F_{drive}=-\frac{\partial^{2}M\left(x,\omega \right)}{\partial x^{2}}=-W\int_{-\frac{d}{2}}^{\frac{d}{2}}E\left(z\right)\beta \left(z\right)\left(z-z_{0}\right)\times \frac{\partial^{2}\Delta \hat{T}\left(x,\omega \right)}{\partial x^{2}}dz$$

The vibration in microcantilever beams is very sensitive to changes in the fluid environment, and it mainly manifests as the resonance frequency and quality factor change significantly with small changes in the operating environment. From the literature [32], cantilever beams are hydrodynamically hindered when vibrating in incompressible fluids. The notation used in the hydrodynamic derivation is defined as the Table 1 shows.

For the hydrodynamic force $F_{hydro}$ is derived as follows:

(29) $$Re=\frac{\rho_{f}\omega D^{2}}{4\eta }$$

(30) $$\Gamma \left(\omega \right)=\Omega \left(\omega \right)\Gamma_{circ}\left(\omega \right)$$

(31) $$\Gamma_{circ}\left(\omega \right)=1+\frac{4iK_{1}\left(-i\sqrt{iRe}\right)}{\sqrt{iRe}K_{0}\left(-i\sqrt{iRe}\right)}$$

(32) $$\Omega \left(\omega \right)=\Omega_{r}+i\Omega_{i}$$

(33) $$\begin{array}{c} \Omega_{r}=\left[\begin{array}{c} 0.91324-0.48274{(\log_{10}Re)}^{1}+0.46842{(\log_{10}Re)}^{2}-0.12886{(\log_{10}Re)}^{3}\\ +0.044055{(\log_{10}Re)}^{4}-0.0035117{(\log_{10}Re)}^{5}+0.00069085{(\log_{10}Re)}^{6} \end{array}\right]\\ \times {\left[\begin{array}{c} 1-0.56964{(\log_{10}Re)}^{1}+0.4869{(\log_{10}Re)}^{2}-0.13444{(\log_{10}Re)}^{3}\\ +0.045155{(\log_{10}Re)}^{4}-0.0035862{(\log_{10}Re)}^{5}+0.00069085{(\log_{10}Re)}^{6} \end{array}\right]}^{-1} \end{array}$$

(34) $$\begin{array}{c} \Omega_{i}=\left[\begin{array}{c} -0.024134-0.029256{(\log_{10}Re)}^{1}+0.016294{(\log_{10}Re)}^{2}-0.00010961{(\log_{10}Re)}^{3}\\ +0.000064577{(\log_{10}Re)}^{4}-0.00004451{(\log_{10}Re)}^{5} \end{array}\right]\\ \times {\left[\begin{array}{c} 1-0.597021{(\log_{10}Re)}^{1}+0.55182{(\log_{10}Re)}^{2}-0.18357{(\log_{10}Re)}^{3}\\ +0.079156{(\log_{10}Re)}^{4}-0.014369{(\log_{10}Re)}^{5}+0.0028361{(\log_{10}Re)}^{6} \end{array}\right]}^{-1} \end{array}$$

(35) $$F_{hydro}=\frac{\pi }{4}\rho_{f}W\omega^{2}\Gamma \left(\omega \right)Z\left(x,\omega \right)$$

where $\Gamma_{circ}\left(\omega \right)$ is the hydrodynamic function of a circular cross-section microcantilever beam. $\Omega \left(\omega \right)$ is the cross-section correction function. It corrects the hydrodynamic function of the circular cross-section to the hydrodynamic function $\Gamma \left(\omega \right)$ of the rectangular cross-section microcantilever beam.

After the resultant force $F_{eff}\left(x,\omega \right)$ of FGM microcantilever beams vibrating in a fluid is derived, we can find its dynamical deformation field $Z\left(x,\omega \right)$ based on Euler–Bernoulli beam theory.

(36) $${\left(EI\right)}_{eff}\frac{\partial^{4}Z\left(x,\omega \right)}{\partial x^{4}}-\rho_{eff}A\omega^{2}Z\left(x,\omega \right)=F_{eff}\left(x,\omega \right)$$

where, ${\left(EI\right)}_{eff}$ is the equivalent bending stiffness of the microcantilever beam. A is the cross-sectional area.

(37) $$\left\{\begin{array}{c} A=W\times h\\ {\left(EI\right)}_{eff}=W\cdot \int_{-\frac{h}{2}}^{\frac{h}{2}}E\left(z\right){\left(z-z_{0}\right)}^{2}dz \end{array}\right.$$

Using Galerkin’s method to solve Equation (36), its solution can be expressed in the form of a free cantilever beam normalized to the vibrational magnitude. In Galerkin’s method, $\varphi_{i}\left(x\right)$ is a trial function determined by the boundary conditions of the cantilever beam. a is the frequency-dependent coefficient to be determined.

(38) $$\varphi_{i}\left(x\right)=a_{i}\left\{\cos\left(k_{i}x\right)-\cosh\left(k_{i}x\right)-\frac{\cos\left(k_{i}L\right)+\cosh\left(k_{i}L\right)}{\sin\left(k_{i}L\right)+\sinh\left(k_{i}L\right)}\left[\sin\left(k_{i}x\right)-\sinh\left(k_{i}x\right)\right]\right\}$$

(39) $$\left\{\begin{array}{c} a_{1}=\frac{1.000000054966522}{\sqrt{L}}\\ a_{2}=\frac{1.0000000424921067}{\sqrt{L}}\\ a_{3}=\frac{1.0000000837026268}{\sqrt{L}} \end{array}\right.$$

(40) $$\left\{\begin{array}{c} k_{1}=\frac{1.875104}{L}\\ k_{2}=\frac{4.694091}{L}\\ k_{3}=\frac{7.854757}{L} \end{array}\right.$$

where $a_{i}\;$is a coefficient determined by the boundary conditions, $k_{i}\;$is the coefficient determined by the order of the trial function. In this paper, we extrapolate to the third order in solving the microcantilever beam vibration pattern. So $a_{i}$ and $k_{i}$ are taken to the third order.

After Equation (36) is solved by Galerkin’s method, the dynamical deformation field $Z\left(x,\omega \right)$ can be expressed as:

(41) $$Z\left(x,\omega \right)=\sum_{n=1}^{\infty }A_{n}\left(\omega \right)\varphi_{i}\left(x\right)$$

(42) $$A\left(\omega \right)=\frac{\int_{0}^{L}F_{drive}\left(x,\omega \right)\varphi_{i}\left(x\right)dx}{{\left(EI\right)}_{eff}\left\{\int_{0}^{L}{\left[\frac{d^{2}\varphi_{i}\left(x\right)}{dx^{2}}\right]}^{2}dx-\frac{\rho_{eff}A\omega^{2}}{{\left(EI\right)}_{eff}}\left[1+\frac{\pi W^{2}\rho_{f}}{4A\rho }\Gamma \left(\omega \right)\right]\right\}}$$

### 2.4. Quality Factor and First Order Resonant Frequency

The quality factor is a characterization parameter of how fast or slow the energy is dissipated in a microcantilever beam during vibration. A high value of quality factor indicates that the microcantilever beam has low energy dissipation during vibration. It retains most of the energy for efficient operation. According to the literature [33], we commonly use the following equation to estimate the quality factor of a cantilever beam operating in a fluid.

(43) $$Q=\frac{{\left(E_{eff}\rho_{eff}\right)}^{\frac{1}{2}}Wh^{2}}{24\eta L^{2}}$$

In Section 2.3 above, we wrote about the process of solving the Euler–Bernoulli equation to obtain the third-order accurate amplitude-frequency response of the FGM microcantilever beam using Galerkin’s method. According to the literature [34], we can estimate the first-order resonant frequency of the microcantilever beam using a less computationally intensive formula as follows:

(44) $$f_{0}=1.875^{2}\times \frac{1}{2\pi L^{2}}\sqrt{\frac{E_{eff}h^{2}}{12\rho_{eff}}}$$

## 3. Example and Result Analysis

In this paper, the mechanical behavior of microscale porous functional gradient materials is analyzed theoretically. However, experimentally, it is not currently possible to fabricate porous functional gradient Cu-Si microcantilever beams with controllable material distribution and pore distribution. We will follow up on the advanced fabrication process at a later time [26,35].

### 3.1. Power-Law Distribution of Material Properties for Porous FGM

To study the power-law distribution of material properties of FGMs with pores, we choose microcantilever beams made of Cu and Si. Its size condition is L=300 μm, W=30 μm, h=10 μm. The upper surface material is Cu, and the lower surface material is Si. n is the gradient factor in the power-law distribution equation. It indicates the percentage of material composition of Cu and Si. The material parameters for Cu and Si are given in Table 2.

**Figure 3:**
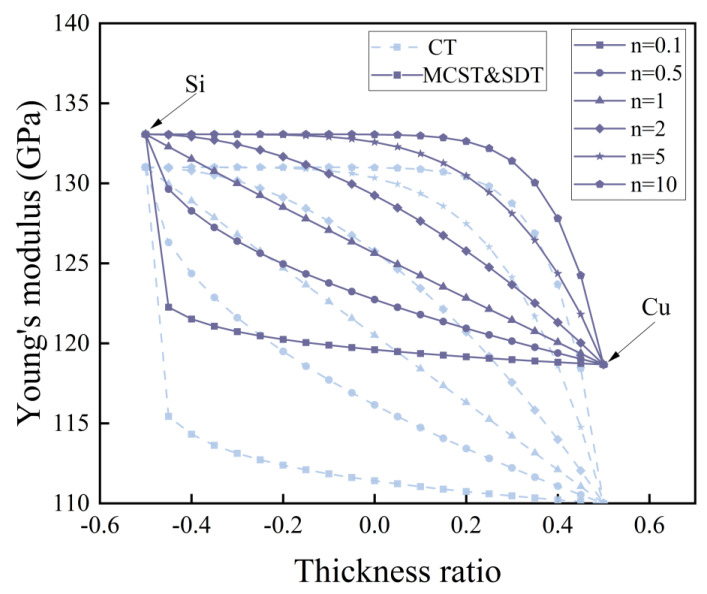
Distribution of Young’s modulus according to thickness ratio for different gradient factors n.

**Figure 4:**
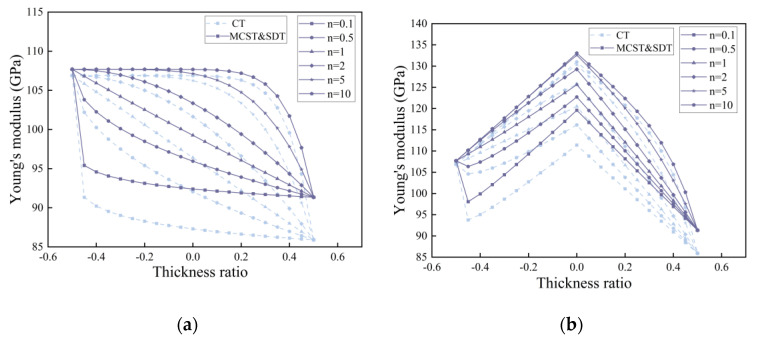

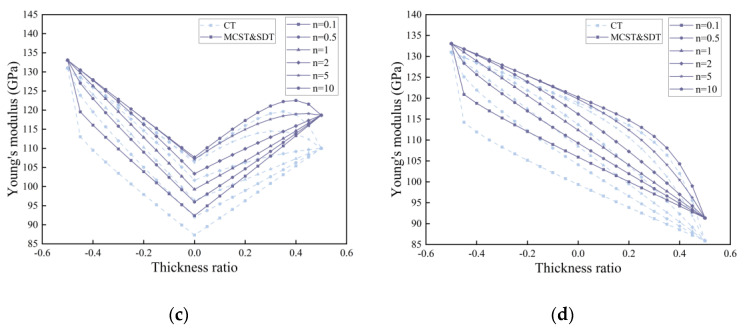
Young’s modulus distribution of microcantilever beams with porosity (α=0.2) at different gradient factors n. (**a**) Even distribution, (**b**) X-type distribution, (**c**) O-type distribution, (**d**) V-type distribution.

Based on the modified couple stress theory (MCST), we have corrected Young’s modulus with the scale constant l, and compared the case without correction in the conventional theory (CT). In Figure 3 and Figure 4, we compare the distribution of Young’s modulus for microcantilever beams without porosity and microcantilever beams with porosity (α=0.2).

In Figure 3, the introduction of the scale effect increases the Young’s modulus of the microcantilever beam. The increase in the gradient factor n leads to an increase in the amount of silicon. Young’s modulus distribution inside the microcantilever beam rises. In Figure 4, the presence of porosity reduces the Young’s modulus of the microcantilever beam as a whole. Different pore distributions have a great influence on the internal Young’s modulus distribution. At higher pore densities, the reduction in Young’s modulus is evident.

**Figure 5:**
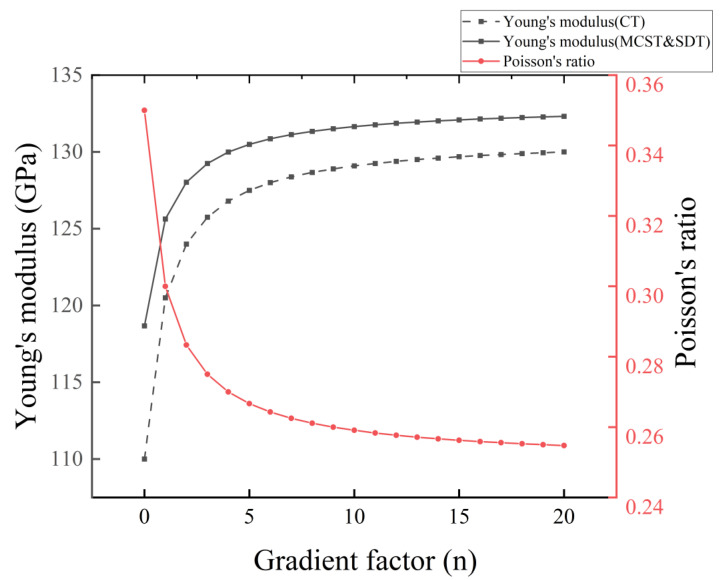
Equivalent Young’s modulus of non-porous microcantilever beams with different gradient factors n.

**Figure 6:**
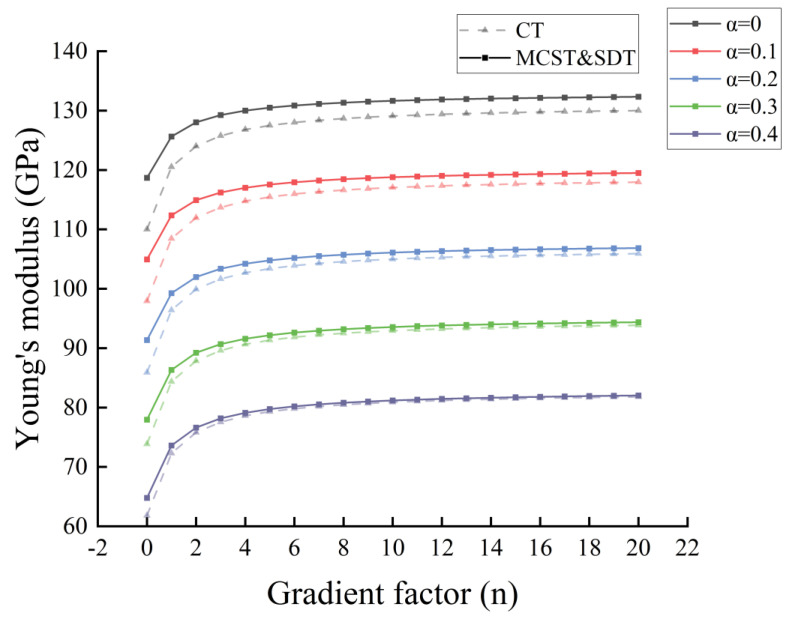
Equivalent Young’s modulus of microcantilever beams with gradient factor at different porosities (Even distribution).

**Figure 7:**
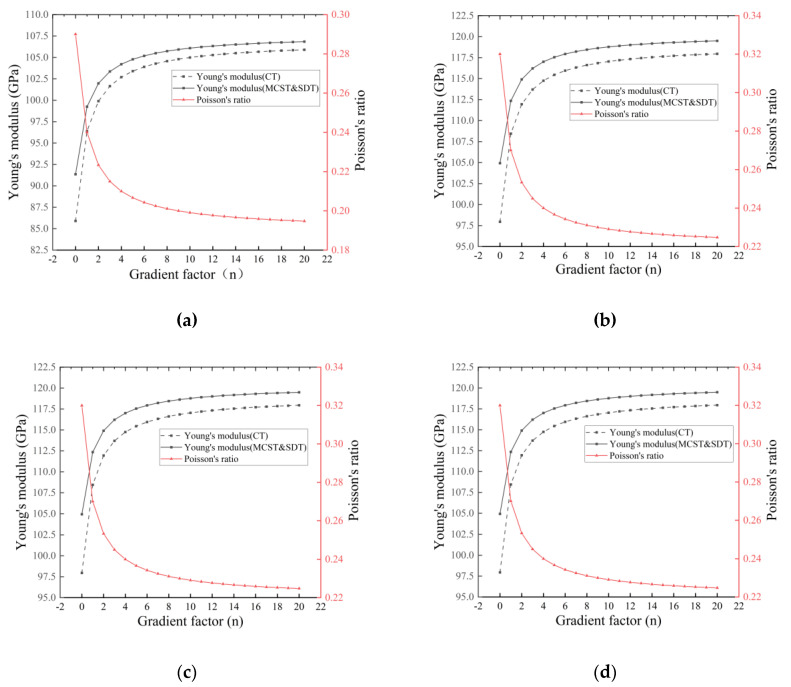
Variation in equivalent Young’s modulus and equivalent Poisson’s ratio with gradient factor n for four pore distributions α=0.2, (**a**) Even distribution, (**b**) X-type distribution, (**c**) O-type distribution, (**d**) V-type distribution.

In Figure 5, we can visualize the correction of the equivalent Young’s modulus based on the scale effect under MCST more intuitively. In Figure 6, the reduction of equivalent Young’s modulus by different porosities is also evident, and the correction of the equivalent Young’s modulus by the scale effect is weakened by an increase in porosity. In Figure 7, the distribution of pores in the interior has little effect on the equivalent Young’s modulus and Poisson’s ratio of the entire microcantilever beam.

### 3.2. One-Dimensional Temperature Field Distribution

**Figure 8:**
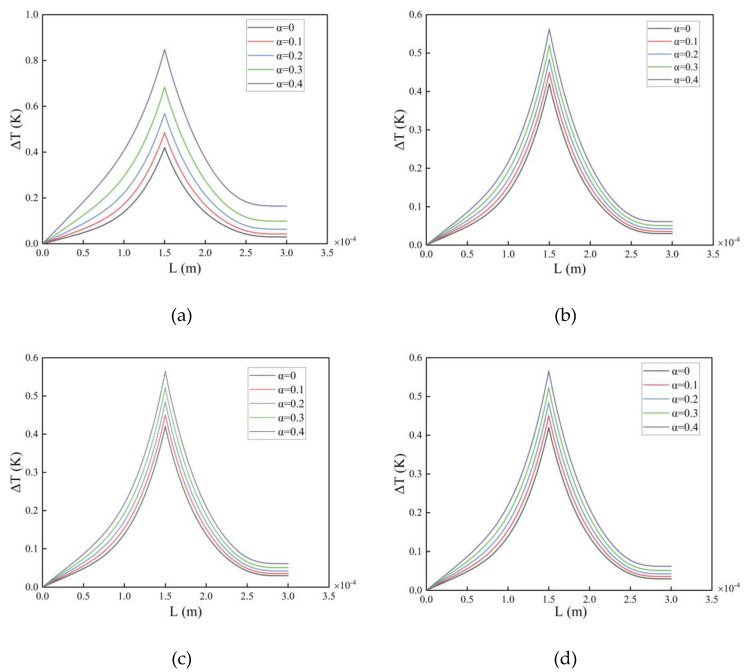
One-dimensional temperature field distributions of microcantilever beams with different porosities in air. (**a**) Even distribution, (**b**) X-type distribution, (**c**) O-type distribution, (**d**) V-type distribution.

In this section, we continue to use the microcantilever beam dimensions from Section 3.1. The laser frequency used is $f_{l}=17,430\;Hz,$ and the power is $P_{0}=0.01\;W$. The loading position of the laser is $x_{0}=0.5\;L$. The convective heat transfer coefficient of a gas to a solid is $\gamma =10\;W/(m^{2}\cdot K)$. The absorption coefficient of laser energy by a microcantilever beam is λ=0.3. The fluid environment in which the microcantilever beam operates is air. Disregarding the effect of scale effect, we investigated the one-dimensional temperature field distribution of microcantilever beams under different porosity conditions separately. The temperature field distribution of the microcantilever beam is shown in Figure 8 below.

The presence of pores produces bubbles inside the microcantilever beam. The one-dimensional temperature field distribution of the microcantilever beam rises with increasing porosity. The uniform distribution of pores has a more pronounced effect on the temperature field than the irregular distribution. The uniform distribution makes the temperature field rise more. There is also a more pronounced linear trend in the rise of temperature with increasing porosity. (b), (c), and (d) three irregular pore distributions have little difference in their effect on the temperature field.

### 3.3. Amplitude-Frequency Response in Fluids

In this section, we use microcantilever beams with dimensions L=2500 μm, W=400 μm, and h=160 μm. The laser loading position is $x_{0}=0.5\;L$. Laser power is $P_{0}=0.5\times 10^{-3}\;W$. The amplitude-frequency response of the FGM microcantilever beams in three different fluids is investigated.

**Figure 9:**
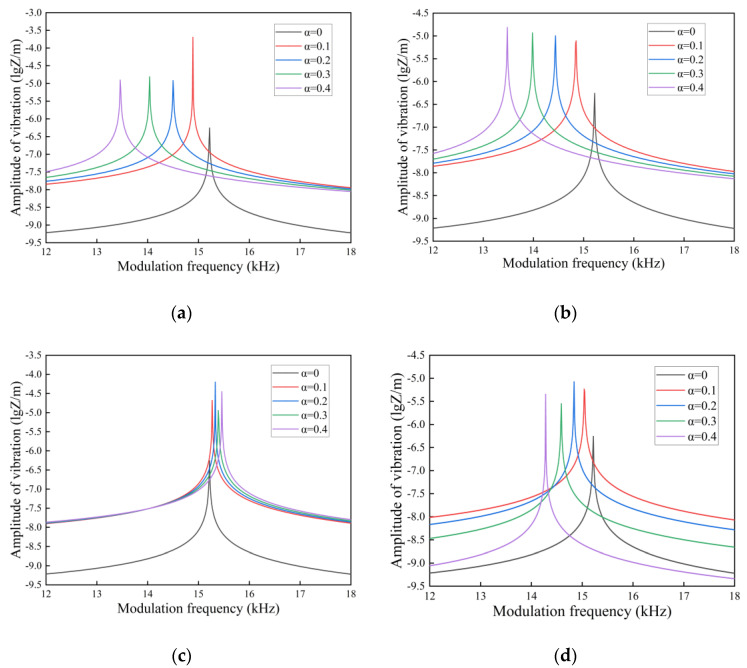
Amplitude-frequency response spectra of FGM microcantilever beams in air with different porosities. ((**a**) Even distribution, (**b**) X-type distribution, (**c**) O-type distribution, (**d**) V-type distribution).

**Figure 10:**
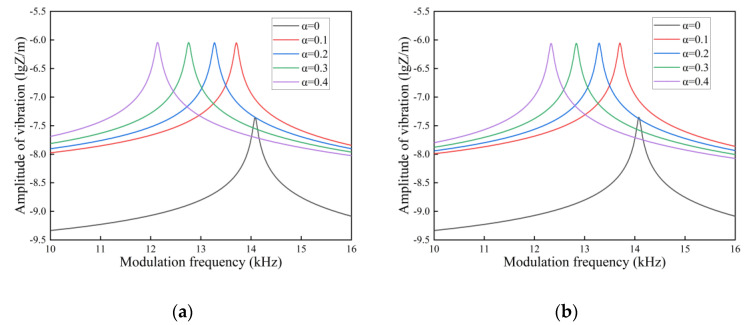

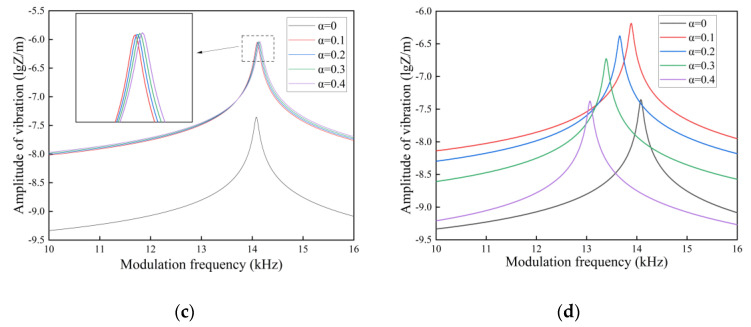
Amplitude-frequency response spectra of FGM microcantilever beams in gasoline with different porosities. ((**a**) Even distribution, (**b**) X-type distribution, (**c**) O-type distribution, (**d**) V-type distribution).

**Figure 11:**
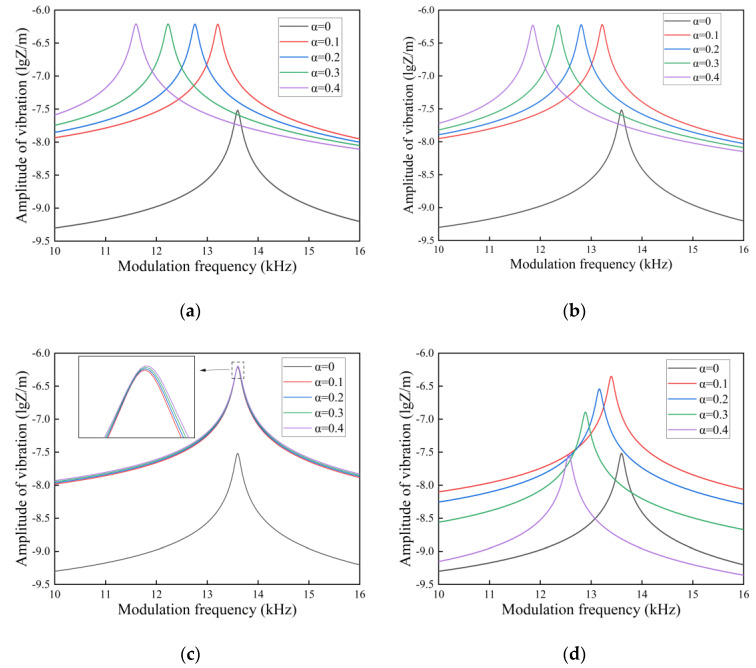
Amplitude-frequency response spectra of FGM microcantilever beams in water with different porosities. (**a**) Even distribution, (**b**) X-type distribution, (**c**) O-type distribution, (**d**) V-type distribution.

In Figure 9, Figure 10 and Figure 11, we investigate the amplitude-frequency response of FGM microcantilever beams with different porosities in air, gasoline, and water. The physical parameters of these three fluids are given in the Table 3.

Comparing Figure 9, Figure 10 and Figure 11 longitudinally, we can find that the resonance summits of the microcantilever beams flatten out as the fluid density and dynamic viscosity increase. This is due to the fact that vibrations in the fluid generate additional damping. The photothermal driving force is partially canceled by the hydrodynamic force. The vibration amplitude of the FGM microcantilever beam also decreases gradually. The amplitude is maximized when vibrating in air, and the amplitude is minimized when vibrating in water.

In a side-by-side comparison, the even distribution of pores and the X-type distribution show a very regular effect. However, the effects of O-type and V-type distributions on microcantilever beam vibrations are well characterized. Due to the presence of pores, the amplitude of the microcantilever beam vibration increases as a whole, and the resonance peaks collectively drift to lower frequencies except for the O-type distribution. The O-type distributed pores give the microcantilever beam a hollow-like structure. This structure causes the internal stress distribution in the cantilever beam to be concentrated at the periphery when the force is applied. It increases the strength of the beam in terms of structure. So, as the porosity increases, the resonance peak of the O-type distribution shifts slightly towards higher frequencies. The V-type distribution allows most of the pores to exist in the Cu portion of the upper material. As the porosity increases, the Cu metal content in the FGM microcantilever beams decreases. The hardness of the microcantilever beam gradually increases when the Si component content is higher than the Cu content. This increase in hardness causes the amplitude of the microcantilever beam to decrease when it vibrates. Therefore, in the V-type distribution, the larger the porosity, the smaller the vibration amplitude will be.

### 3.4. Quality Factor and First Order Resonant Frequency

In this section, we investigate the quality factor of the FGM microcantilever beam with L=300 μm and W=30 μm vibrating in three fluids. The gradient factor n of the FGM microcantilever beam was determined to be 0.1, and the thickness h was increased from 50 μm to 300 μm. The porosity was used as a study parameter.

**Figure 12:**
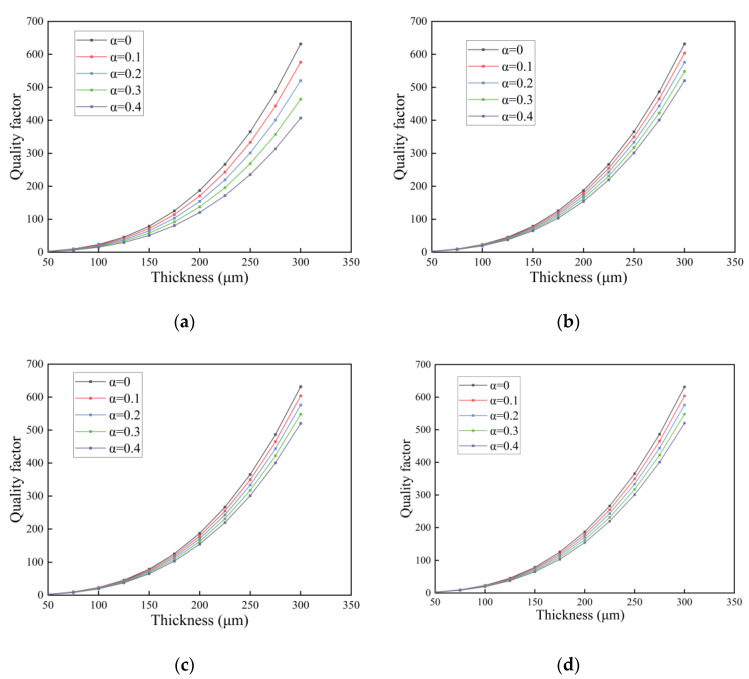
Variation in quality factor with thickness in air for FGM microcantilever beams with different porosities. (**a**) Even distribution, (**b**) X-type distribution, (**c**) O-type distribution, (**d**) V-type distribution.

**Figure 13:**
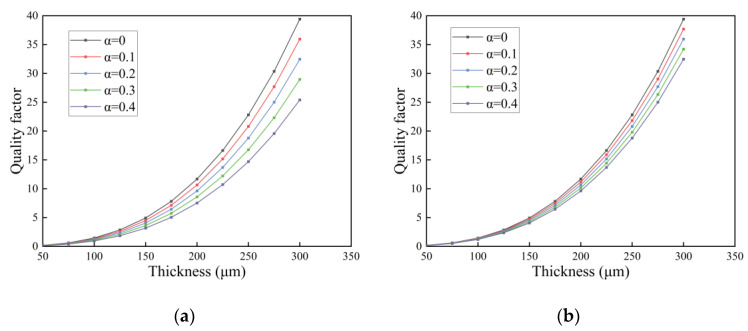

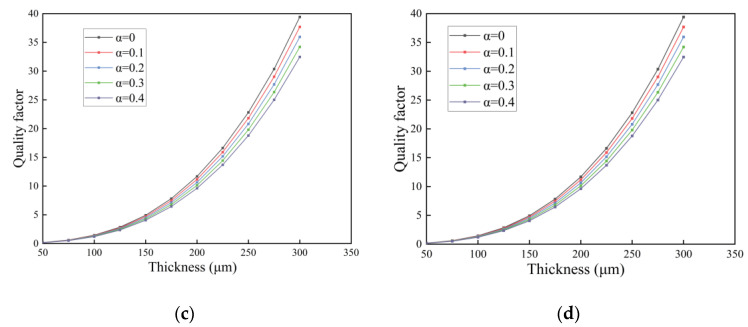
Variation in quality factor with thickness in gasoline for FGM microcantilever beams with different porosities. (**a**) Even distribution, (**b**) X-type distribution, (**c**) O-type distribution, (**d**) V-type distribution.

**Figure 14:**
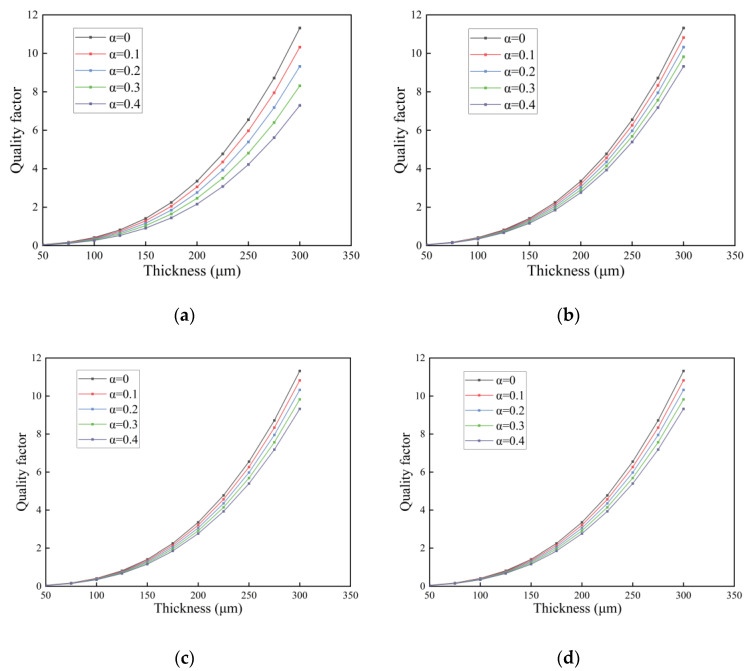
Variation in quality factor with thickness in water for FGM microcantilever beams with different porosities. (**a**) Even distribution, (**b**) X-type distribution, (**c**) O-type distribution, (**d**) V-type distribution.

In Figure 12, Figure 13 and Figure 14, the quality factor is clearly affected by the fluid environment. In a fluid environment with high density and dynamic viscosity, the quality factor of the FGM microcantilever beam is very small, and energy loss during vibration is very high. The presence of pores resulted in a slight decrease in the quality factor of the microcantilever beams. This is due to the fact that the pores cause a decrease in the Young’s modulus of the microcantilever beam. The quality factor of even distributed microcantilever beams is more sensitive to the response of pores. The quality factor decreases faster with increasing porosity. The other three non-uniformly distributed microcantilever beams show a small decrease. We provide data on some of the quality factors, as detailed in the Appendix A.

**Figure 15:**
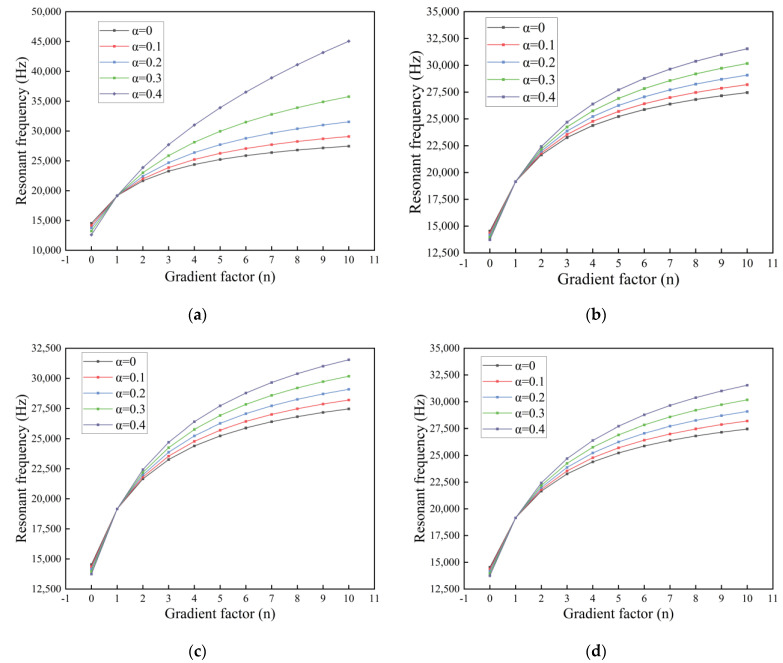
First-order resonant frequency of an FGM microcantilever beam in air with gradient factor changes. (**a**) Even distribution, (**b**) X-type distribution, (**c**) O-type distribution, (**d**) V-type distribution.

In Figure 15, for the first-order resonant frequency of the microcantilever beam in air, we use the same microcantilever beam dimensions as in Section 3.3. The gradient factor is n=0.1.

**Figure 16:**
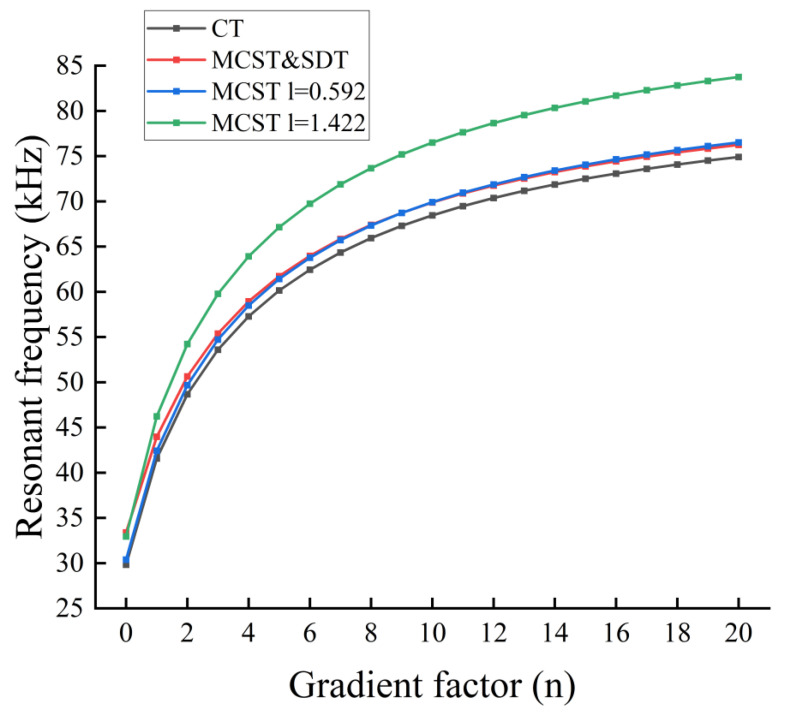
Resonant frequencies of FGM microcantilever beams with different gradient factors. (Even distribution, α=0.2).

From the Figure 16, we can see that porosity causes a decrease in the first-order resonant frequency. The effect of porosity on the first-order resonance frequency of even distributed microcantilever beams is large but small for non-uniformly distributed microcantilever beams. Corrective even stresses are considered to have a gain effect on the resonance frequency of the microcantilever beam. The larger the scale constant l is, and the scale thickness ratio l/d decreases, the more pronounced the gain effect of MCST & SDT.

## 4. Conclusions

In this paper, based on the Euler–Bernoulli beam theory and modified couple stress theory, the one-dimensional heat conduction equation of functional gradient beams under photothermal excitation is established. Based on the physical neutral plane theory, the temperature field distribution of the FGM microcantilever beam is obtained. By introducing the hydrodynamic function, the analytical expressions of the photothermal vibration model and the dynamic deformation field of the FGM microcantilever beam in fluid are obtained. The effects of scale effect, porosity, geometry, material gradient factor, and fluid environment on the photothermal vibration in the functional gradient beam are investigated by numerical calculations. We can draw the following conclusions:

(1) The scale effect increases the Young’s modulus of the FGM microcantilever beam. On the contrary, the presence of pores decreases Young’s modulus. The greater the porosity, the greater the decrease in Young’s modulus. The pores attenuate the correction of Young’s modulus by the scale effect. FGM microcantilever beams with large porosity are less affected by the scale effect. In engineering, we can control the porosity and pore distribution to attenuate the scale effect at the microscale.

(2) The presence of pores causes the one-dimensional temperature field of the FGM microcantilever beam to rise. The extent of the effect is highly dependent on the distribution of pores. When the porosity increases, the even distribution is affected more than the non-uniform distribution. The pores cause the amplitude-frequency response of the microcantilever beam to drift to lower frequencies and increase in amplitude as a whole. The distribution of the pores also has a significant effect on the amplitude-frequency response, where the amplitude-frequency response is characterized differently by the O-type and V-type distributions.

(3) The quality factor of FGM microcantilever beams is strongly influenced by the fluid environment. The presence of pores causes a small decrease in the quality factor. The larger the porosity, the more the quality factor decreases, and the faster the energy is dissipated when the cantilever beam vibrates. In contrast to the even distribution, the quality factor of non-uniformly distributed microcantilever beams is less affected by the pore space.

## Figures and Tables

**Table 1:** Table of parameters used in hydrodynamic derivation.

Parameter Name	Symbolic	Parameter Name	Symbolic
Reynolds number	*Re*	fluid dynamic viscosity	η
fluid density	$\rho_{f}$	Type III Bessel Functions	$K_{0},\;K_{1}$
Diameter of circular section	D		

**Table 2:** The material parameters for Cu and Si.

Material Parameters	Cuprum (Cu)	Silicon (Si)
E (GPa)	110	131
$\rho \;(kg/m^{3})$	8900	2330
v	0.35	0.25
l (μm)	1.422	0.592
$\kappa \;(W\cdot {\left(m\cdot K\right)}^{-1})$	401	150
C(J/(kg·K))	386	695
$\beta \;(\times 10^{-6}\cdot K^{-1})$	16.5	3

**Table 3:** Fluid Density and Dynamic Viscosity.

Fluids	$\rho \;(kg/m^{3})$	η (Pa·s)
air	1.205	$1.81\times 10^{-5}$
gasoline	678	$2.9\times 10^{-4}$
water	998	$1.01\times 10^{-3}$

## Data Availability

Data are contained within the article.

## Appendix A

**Table A1:** Quality factors of FGM microcantilever beams in three fluids. (Even distribution, d=200 μm).

Fluids	α	Quality Factors
air	0	187.12
	0.1	170.68
	0.2	154.14
	0.3	137.46
	0.4	120.59
gasoline	0	11.68
	0.1	10.65
	0.2	9.62
	0.3	8.58
	0.4	7.53
water	0	3.35
	0.1	3.06
	0.2	2.76
	0.3	2.46
	0.4	2.16

**Table A2:** First-order resonant frequencies of FGM microcantilever beams in air. (Even distribution).

n	α	Resonant Frequencies (Hz)
1	0	19,155.38
	0.1	19,155.38
	0.2	19,155.38
	0.3	19,155.38
	0.4	19,155.38
5	0	25,228.81
	0.1	26,255.51
	0.2	27,712.75
	0.3	29,956.38
	0.4	33,911.87
10	0	27,459.22
	0.1	29,084.01
	0.2	31,542.55
	0.3	35,758.53
	0.4	45,056.91
